# Spatial Distribution Patterns of *Parthenolecanium corni* (Hemiptera, Coccidae) and of the Ampelovirus GLRaV-1 and the Vitivirus GVA in a Commercial Vineyard

**DOI:** 10.3390/v12121447

**Published:** 2020-12-16

**Authors:** Gérard Hommay, Louis Wiss, Catherine Reinbold, Joël Chadoeuf, Etienne Herrbach

**Affiliations:** 1 Université de Strasbourg, Institut National de Recherche pour l’Agriculture, l’Alimentation et l’Environnement (INRAE), Unité Mixte de Recherche Santé de la Vigne et Qualité du Vin (SVQV), F-68000 Colmar, France; catherine.reinbold@inrae.fr (C.R.); etienne.herrbach@inrae.fr (E.H.); 2 Institut National de Recherche pour l’Agriculture, l’Alimentation et l’Environnement (INRAE), Unité de Recherche Biostatistique et Processus Spaciaux (BioSP), F-84914 Avignon, France; joel.chadoeuf@inrae.fr

**Keywords:** soft scale, grapevine leafroll disease, grapevine virus A, spatial distribution, virus propagation

## Abstract

Distribution patterns of the European fruit lecanium *Parthenolecanium corni* (Bouché) and of grapevine leafroll-associated virus-1 (GLRaV-1) and grapevine virus A (GVA) were monitored from 2003 to 2015 in a Riesling vine plot in the northeast of France. Virus spread was compared between two periods: 2003–2008 and 2009–2014. The percentage of infected vines increased from 54 to 78% for GLRaV-1 and from 14 to 26% for GVA. The spatial distribution of viruses and of *P. corni* was analysed using permutation tests and revealed an aggregative pattern. Virus distribution was not associated with the density of *P. corni* population on grapevines. However, GLRaV-1 and GVA spread mainly from initially infected vines. New GLRaV-1 and GVA infections were more frequent on vines near primarily infected vines, first anisotropically along the row, then between neighbouring rows. Virus spread was similar to those described in literature with grapevine mealybug species. This slow vine-to-vine progression suggests that *P. corni* was responsible for the virus spread, in accordance with the low mobility and low transmission capacities of its local population.

## 1. Introduction

Progressive reduction of insecticide use in vineyards, changes in farming practices, and increasing commercial exchanges have favoured the outbreak of scale insect (Hemiptera, Coccoidea) populations. Mealybugs (Pseudococcidae) and soft scales (Coccidae) dwelling on grapevine were for a long time considered as secondary pests, until they were shown to transmit grapevine leafroll-associated viruses (GLRaVs) in different winegrowing regions of the world [1,2]. At least five serologically distinct members of the Closteroviridae family, designated as GLRaV-1, -2, -3, -4, and -7, are associated with leafroll disease [3,4,5]. Three of these viruses (GLRaV-1, -3, and -4), which belong to the *Ampelovirus* genus, are transmitted by several species of mealybugs and soft scales [1]. Moreover, these vectors are able to inoculate three grapevine-infecting viruses associated with “rugose wood complex”: grapevine virus A (GVA), GVB, and GVE [1] assigned to the genus *Vitivirus* (family Betaflexiviridae). 

Pietersen [6] and Cabaleiro [7] identified three main types of epidemic for leafroll disease: first, planting of leafroll-infected material which is usually distributed at random among healthy plants in vineyards; secondly, transmission of leafroll viruses by vectors from infected to neighbouring healthy plots; and lastly, virus propagation within a vine plot by vectors from initially infected plants. In addition, the spatiotemporal pattern of spread was assigned to root grafting at least in one case reported in Spain [8].

The distribution of virus-infected vines is generally random when planting material is infected at a low incidence but tends to have an even distribution when this material contains a high rate of infected vines [9]. Occurrence of random clusters of infected vines depends on the process of producing material and whether infected cuttings remain associated when planting. Studies on grapevine leafroll disease (GLRD) dissemination by scale insects within a vineyard showed that in the first years, distribution of infection was random and afterwards became aggregative [10,11]. Sokolsky et al. [12] reported three stages in spatiotemporal infection spread: a first main random spread, then aggregated spread along adjacent vines, and lastly a more uniform spread within the vineyard. Studies on spatiotemporal distribution patterns of leafroll-diseased vines often demonstrated a high degree of aggregation, consistent with a vector propagation of the pathogen [10,13,14,15,16]. It has been demonstrated that, although mealybug species are unable to fly (apart from adult males, which are not vectors), some species rapidly spread the GLRD from infected plots to new adjacent plantations [8,11,13,14,17,18].

Systematic removal of diseased vines combined with insecticide treatments applied after the hatching of crawlers (first-instar larvae) resulted in significant decrease of viral extension [9,19]. In New Zealand, Bell et al. [20] showed that removing GLRaV-3-infected vines reduced and maintained incidence under 1%. Roguing was shown to be economically viable if disease prevalence was less than 25% [21].

Scale insects have several ways of dispersal [22]. In the vineyard, nymphs crawl easily from vine to vine on their interweaved foliage. Nymphs can also be transported by the winegrowers and their implements during the different winegrowing works. Finally, scale-attending ants that carry nymphs may also contribute to the spread of virus [23,24,25,26]. These ways lead predominantly to within-row transmission and aggregative distribution. Passive dispersal by other animals (phoresy) such as mammals, birds, or insects is not documented in the vineyard. The main way of crawler dispersal in the vineyard is the wind [27,28,29,30]. In a uniform landscape, wind dispersal would theoretically lead to a random deposition of crawlers onto vines. In addition, wind dispersal of fallen leaves bearing larvae is also possible [31]. Thus, the spread of GLRD by scale insects may involve a combination of natural crawling, passive transport, and wind dispersal [11,32].

The coccid *Parthenolecanium corni* (Bouché) is a soft scale widespread in vineyards of Eurasia and North America. In the northern wine-growing regions of Europe, *P. corni* has one generation per year, whereas in southern regions it can develop two or three generations [33,34,35,36]. This species is able to transmit GLRaV-1, GLRaV-3, as well as GVA [37,38,39,40], but its field dispersal and its role on virus spread have not been documented thus far. In northeastern France, *P. corni* is one of the most common scale insect species in vineyards. In this region, GLRaV-1 is the most prevalent virus, followed by GLRaV-3 [41,42]. As GLRD causes important losses of wine production and quality worldwide, it is important to evaluate the role of *P. corni* in disease spread. Therefore, we monitored the evolution of the spatial distribution of *P. corni* and the virus transmitted, GLRaV-1 and GVA, in a commercial vine plot to better assess the risk of field spread of leafroll disease by this soft scale. These observations could contribute to the management strategy against the expansion of the disease and on the choice of pest control means, e.g., roguing either diseased vines only or the entire plot, protective barriers from neighbouring contaminated vines that could require a collective involvement of winegrowers on the same hillside.

## 2. Materials and Methods

The vine plot, under organic pest management for over 20 years, is planted with cv. Riesling and located at Nothalten (Bas-Rhin, Alsace, France; latitude 48°21′31″ N, longitude 07°24′40″ E, altitude 270 m). It consists of 10 rows of 68 to 76 stocks south–north orientated along the slope. The 5 first pathways between rows are around 2.10 m wide, while the 4 following ones are 2.80 m. Stocks are spaced of 1.40 m within rows, except on row 6 where the 7 last vines are closer. Vine management remained the same over the monitoring period, except that since 2007 green manure replaced natural soil cover of the inter-rows. This plot shows a high density of *P. corni*, and a few individuals of *Pulvinaria vitis* (L) and *Heliococcus bohemicus* Sulc. Out of 704 vines tested, 281 were negative; GLRaV-1 is the main virus present (389 vines), either alone or in association with GLRaV-2 (10 vines), GLRaV-3 (110 vines), and GVA (138 vines). The vine plot is surrounded by other vine plots much less infested by the same species of scale insects and infected by the same viruses [30]. The old eastern plot was uncultivated over a few years, then planted in 2008 with new vines; this plot was studied for aerial dispersal of *P. corni* crawlers [29,30].

### 2.1. Mapping Viruses in the Plot

Infection of each stock was checked by double-antibody sandwich enzyme-linked immunosorbent assay (DAS-ELISA). Tissue extracts were obtained from pooled fragments of leaves from several twigs sampled from July to October, or from dormant canes. Leaf or cane fragments (1 g leaves or 0.5 g wood for 5 mL buffer) were ground inside extraction bags with a bullet blender (Homex 5, Bioreba, Reinach, Switzerland). Polyclonal antibodies raised against GLRaV-1, -2, or -3 or GVA were produced in the laboratory and used in a biotine-streptavidine procedure [43]. Absorbance was recorded at 405 nm using a multiscan microplate reader (Thermo Labsystems, Helsinki, Finland). The distribution of viruses was entirely mapped in the plot, within 2 periods, 2003–2008 and 2009–2014. Every vine was tested once at each observation period, with some vines tested several times as they were used in infectivity tests [44]. Additional reverse transcription polymerase chain reaction (RT-PCR) tests were made for grapevines for which ELISA results were not clear-cut. Virus detection was completed by RT-PCR tests on leaves, dormant canes, or soft scales for 65 of the vines. Multiplex RT-PCR for the detection of GLRaV-1, -2, and -3 and GVA was performed using the primers and the protocol developed in our laboratory [45]. PCR products were visualised under UV light on a 2% agarose gel stained with ethidium bromide.

### 2.2. Distribution of Soft Scales

Every spring from 2006 to 2015, between the last week of April and the first week of June, the number of young females of *P. corni* was counted on each stock on the whole plant. From 2003 to 2005, spring counts were taken from studies done by Kuntzmann [46,47,48]—population densities were divided into three categories: absent, 1 to 10, and over 10 adult females per vine. The spatial distribution for each year was represented on grid maps, where each vine was featured by a square and coloured according to infestation level. To test if adult females were independently distributed in the vine plot, we performed permutation tests [49] based on 3000 independent random permutations of female numbers among living stocks. 

### 2.3. Distribution of Viruses

**Figure 1:**
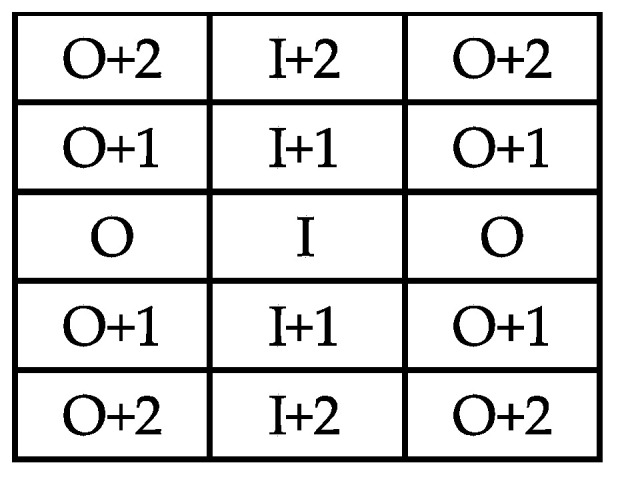
Diagram of the positions of the 14 neighbouring vines distributed around an infected vine (I). I + 1: first neighbour within the same row, I + 2: second neighbour within the same row, O: opposite vine within the next row, O + 1: first neighbour of the opposite vine within the next row, O + 2: second neighbour of the opposite vine within the next row.

Distribution of viruses was mapped for each stock over 2 periods: 2003–2008 and 2009–2014. New infected vines around an initially infected vine (I) were split into 6 categories: first (I + 1) and second (I + 2) next vines in the row, and opposite (O, O + 1, and O + 2) vines located on the two immediate neighbouring rows (Figure 1). Other positions represented infected vines situated beyond this surrounding belt.

On the basis of previous studies [6,8,10,12,50], we assumed that virus spread decreased with distance to an infected vine and was higher within rows than across rows. Numbers of new infected vines were distributed according to the following ranking: first vines along the same row, nearest opposite vines, second neighbours of the same row, then first and second neighbours of the opposite vines (I + 1 > O > I + 2 > O + 1> O + 2), and lastly on other positions. Vines that belonged to more than one group were recorded into the group with the higher expected infection risk. The number of new infected vines observed around I vines was compared to the random proportion of their total number for each position (2*I + 1, 2*I + 2, 2*O, 4*O + 1, 4*O + 2) by a chi^2^ test.

### 2.4. Statistical Tools

For each mapping year, we performed permutation tests for spatial independence to assess whether young *P. corni* females and viruses were clustered in aggregates or randomly distributed within the plot. The spatial dependence between virus-infected vines was also tested for the 2 monitoring periods. As the local population of *P. corni* did not transmit GLRaV-3 in our experiments, this virus did not extend its distribution; therefore, we tested distribution of the whole infected vines over the survey period (2003 to 2014).

Spatial analyses were conducted using procedures designed by Peyrard et al. [49] and operating on R Development Core Team software (version 2.13.0) [51]. Monte Carlo tests were conducted to study the distribution of young females and viruses. Each test was performed with 3000 permutations for distances increasing from 1 to 10 vine spacings. We used the variogram along the row as statistical function of the distance between neighbouring plants (1.40 m) as unit lag. Dead plants were considered as missing data and were not permuted. The null hypothesis of each test was rejected when variogram was outside the confidence interval delimited by Monte Carlo confidence levels of 2.5% and 97.5% obtained under the independence assumption [49]. 

We computed the probability to find a vine infected by a given virus between 2009 and 2014 as a function of distance from a vine contaminated between the years 2003–2008.

To detect the effect of a delay between virus inoculation and symptom development, we calculated the probability of virus presence on each vine during the period of 2009–2014 as a function of yearly *P. corni* female numbers for 2009 to 2013.

### 2.5. Meteorological Records

Daily records of temperatures (°C), total precipitation (L/m^2^), and wind speed (km/h) were obtained from a meteorological station (La Crosse Technology WS 3600) located in the vineyard of Kintzheim, a village located at about 12 km south of the experimental plot (latitude 48°15′00″ N, longitude 07°23′48″ E, altitude 198 m).

## 3. Results

### 3.1. Distribution of P. corni Young Adult Females

**Figure 2:**
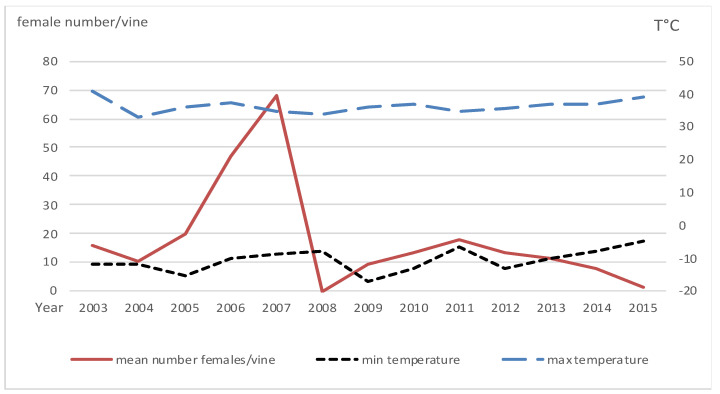
Left *y*-axis: mean number of young females of *Parthenolecanium corni* per vine, from 2003 to 2015 in the Riesling plot in Nothalten. Right *y*-axis: Extreme minimal --- and maximal ^___^^___^ temperatures (in °C) recorded in Kintzheim (12 km south of the study plot).

The population density of *P. corni* females increased from 2004 to 2007, then showed a steep drop in 2008 and a slight increase until 2012 to fall again until 2015 (Figure 2). High summer temperatures recorded in 2003 (Figure 2) were not related to population levels, and the latter declined in 2008 before the occurrence of the heaviest frost of the study period in 2009. In October, minimal temperature fell precociously to 5 °C on 8th 2007, to 3 °C on 5th 2008 and to 5 °C on 3rd 2009, which could have contributed to the decline of *P. corni* populations (Table 1). During the monitoring years, no peculiar event of heavy rain occurred during the period of crawler dispersal (June–July).

**Figure 3:**
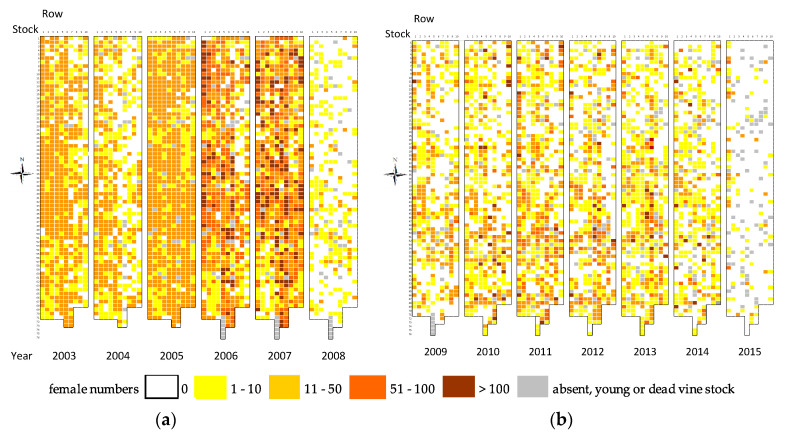
Evolution of the spring distribution of young females of *Parthenolecanium corni* from 2003 to 2014 in the Riesling plot in Nothalten. Each square corresponds to a single vine stock. (**a**) From 2003 to 2008. (**b**) From 2009 to 2015. The *x*-axis represents row numbers, whereas vine stocks within rows are numbered along the *y*-axis, with numbers increasing from north to south.

Figure 3 displays successive distribution maps of young females from 2003 to 2015. Density of *P. corni* was very variable between vines, with a maximum of around 200 females per vine. *P. corni* was spread throughout the plot, with a lesser density mainly on the northeast border, then on the southwest border (Figure 3). Mapping showed successive expansion and regression of colonised zones from the most densely populated vines. Since the start of annual surveys of *P. corni* on each stock in 2003, only one plant (row 5, vine 71) seemed to never have been colonised by this species.

**Figure 4:**
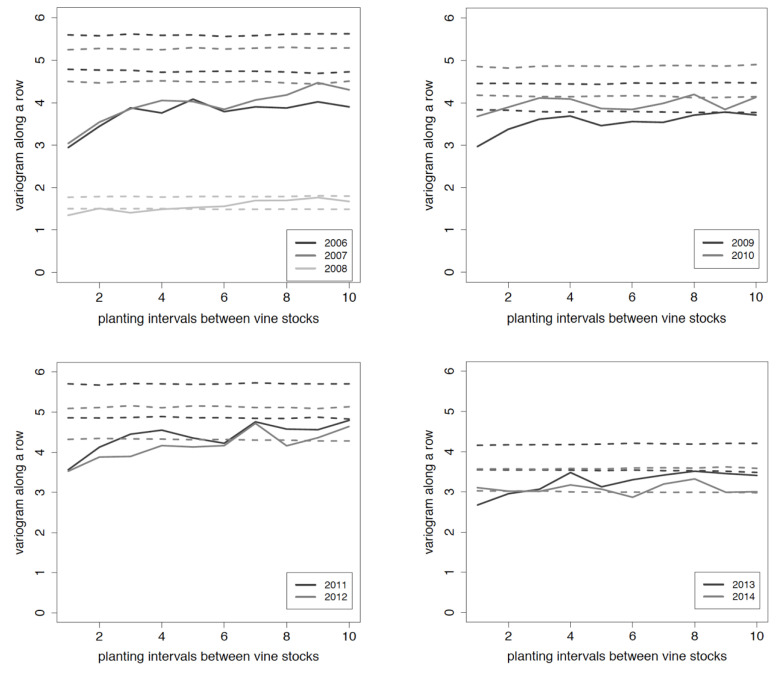
Estimated variograms and their confidence bands generated by permutation tests for spatial distribution of young females of *Parthenolecanium corni* in the Riesling plot. Solid lines represent variogram on *P. corni* numbers per vine. Dotted lines delineate the confidence bands at 95%. A spatial dependence among data is proven when the variogram (solid line) is outside the confidence band. *x*-axis: planting intervals between vine stocks; *y*-axis: value of the variogram along a row.

The statistical spatial analysis of soft scales colonisation enabled us to assess each year the aggregated patterns of *P. corni* infestation (Figure 4). Monte Carlo permutation tests showed a dependent spatial repartition of females on distances up to 4 and 10 planting intervals along the row where aggregation occurred.

### 3.2. Distribution of Viruses

**Figure 5:**
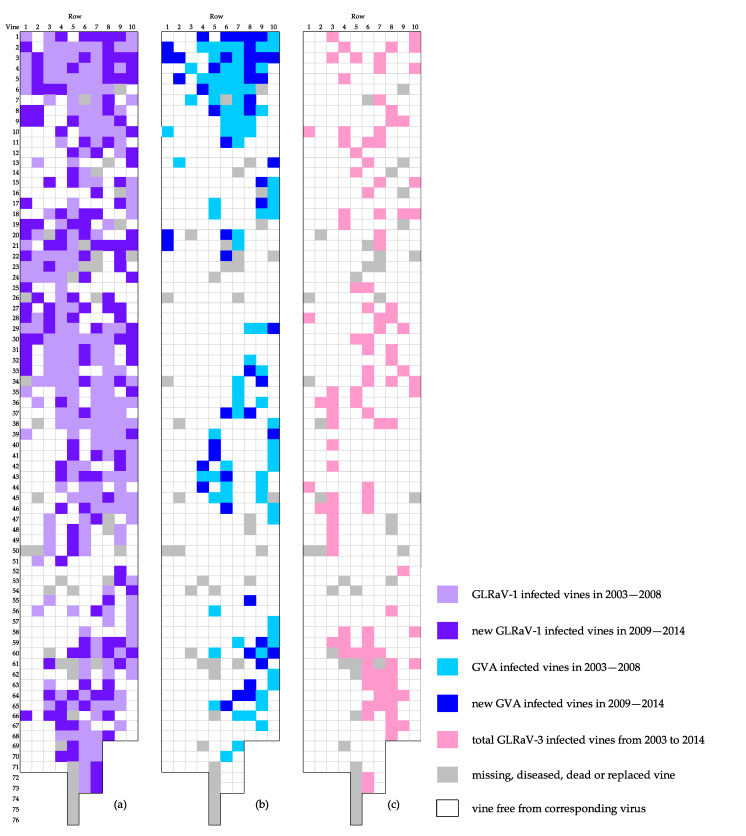
Evolution of the spatial distribution of grapevine leafroll-associated virus-1 (GLRaV-1) (**a**) and of grapevine virus A (GVA) (**b**), from 2003 to 2014 in the Riesling plot in Nothalten. (**c**) Spatial distribution of all GLRaV-3-infected vines over the survey period 2003–2014. Each square corresponds to a vine stock. The *x*-axis represents row numbers, whereas vine stocks within rows are numbered along the *y*-axis, with numbers increasing from north to south.

Among a total of 704 initial living stocks, some could not be tested by ELISA or RT-PCR at each period (2003–2008 and 2009–2014) because of mortality or replacements occurring in the meantime. Out of the 682 remaining stocks, 419 (62%) were initially infected by either one or a combination of the two viruses GLRaV-1 and -3. GVA was always present in mixed infection with GLRaV-1 and/or -3, except for one vine. Only 10 vines were positive for GLRaV-2, alone or in combination with one to three of the aforementioned viruses. Distributions of GLRaV-1 and of GVA are plotted for the vines that survived over the periods 2003–2008 to 2009–2014 (Figure 5a,b). Distribution of GLRaV-3 did not evolve between the two periods, except changes due to mortality and replacement of isolated vines (Figure 5c). Between the periods 2003–2008 and 2009–2014, the number of plants positive for GLRaV-1 raised from 234 to 389, and those positive for GVA increased from 87 to 138. Distribution of newly contaminated vines progressed mainly from the edges of previous infected zones (Figure 5). Thus, the percentage of infected plants grew from 54 to 78% for GLRaV-1 and from 14 to 26% for GVA.

Mapping of GLRaV-1 spread showed that within-row vines immediately next to an infected vine (‘first’ vines) were the most infected, then opposite vines, second neighbours within-row, and first and second vines near the opposite vines (Table 2). Only eight new infected vines (among 155) were observed beyond this perimeter. Moreover, five out of these were situated on the border rows, possibly in the vicinity of GLRaV-1-infected vines in neighbouring plots. GVA spread to within-row vines immediately near an infected vine, then to opposite vines, second vines within-row, and first vines near the opposite vines. Only five new infected vines (among 51) were observed beyond this perimeter, of which four were located on the western border rank, possibly in the vicinity of infected vines. The most remote infected vines from a primary infected vine were 8.4 m for GLRaV-1 and 9.4 m for GVA.

Numbers of new GLRaV-1- and GVA-infected vines around initial ones were significantly different from the numbers of neighbours distributed among the five possible positions around an infected vine (respectively chi^2^ = 84.1, d*f* = 4, *p* < 0.001; chi^2^ = 29.7, d*f* = 4, *p* < 0.001).

**Figure 6:**
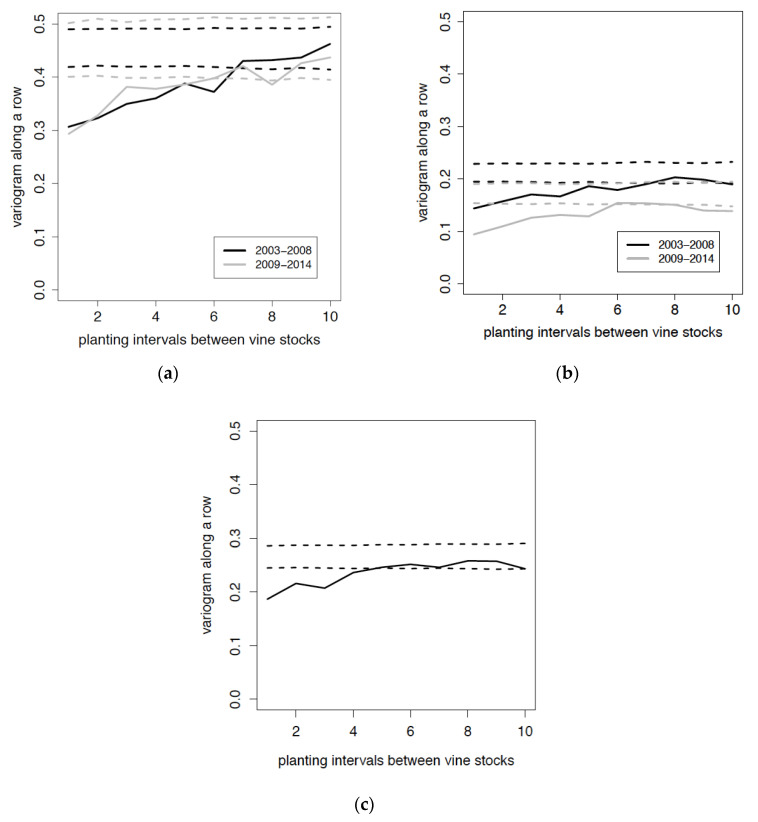
Estimated variograms and their confidence bands generated by permutation tests for spatial distribution of virus-infected vines in the Riesling plot. (**a**) GLRaV-1, (**b**) GVA. Black solid line: variogram on infected vines in 2003–2008. Grey solid line: variogram on infected vines in 2009–2014. (**c**) GLRaV-3 during the whole period 2003–2014. Dotted lines delineate the confidence bands at 95%. A spatial dependence between data is proven when the variogram (solid line) is outside the confidence band. *x*-axis: planting intervals between vine stocks; *y*-axis: value of the variogram along a row.

The variograms calculated on the spatial distributions of GLRaV-1 and of GVA infected vines in 2003–2008 and 2009–2014 revealed an aggregative pattern (Figure 6a,b). Permutations by rotation within the row proved a significant aggregation along the row. Figures show a significant aggregation of GLRaV-1 and GVA at distances of up to six vine spacings in 2003–2008 and up to five vine spacings in 2009–2014. The variogram calculated on the spatial distribution of GLRaV-3 on the total of vines infected over the survey period 2003–2014 also showed a significant aggregation at distances of up to four vine spacings (Figure 6c).

**Figure 7:**
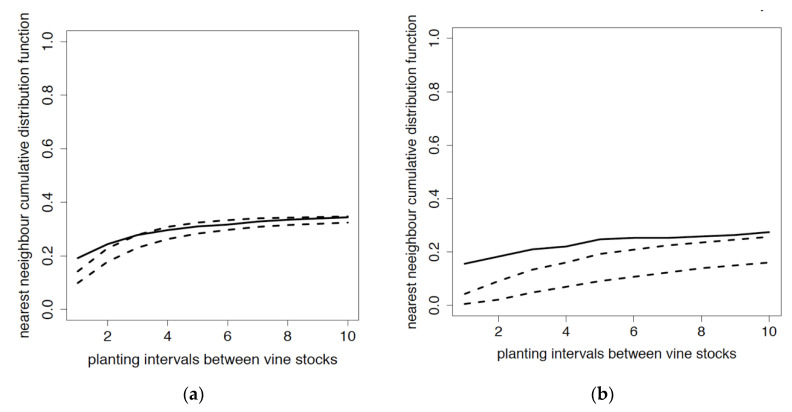
Cumulative distribution function of the distance of a vine infected in 2009–2014 to its nearest neighbour infected in 2003–2008. (**a**) GLRaV-1, (**b**) GVA. Spotted lines delineate the confidence bands at 95% of the cumulative distribution under independence assumption. A spatial dependence between data is proven when probability to find the nearest neighbour at a distance less than *X* (solid line) is outside the confidence band. *x*-axis: planting intervals between vine stocks; *y*-axis: probability to find the nearest neighbour at a distance less than *X*.

The spatial dependence between GLRaV-1- and GVA-infected vines in the two periods 2003–2008 and 2009–2014 was tested (Figure 7a,b). For GLRaV-1, it was significant until two vine spacings, indicating a single vine-to-vine dispersal of this virus. For GVA, dependence on initial infected vines was observed for up to 10 vine spacings. 

### 3.3. Relation between Viruses and Mealybug Distributions

**Figure 8:**
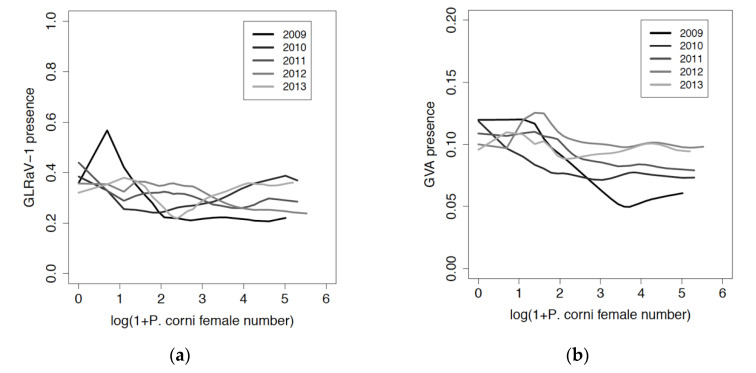
Relation between virus presence in 2009–2014 in vines and female numbers of *Parthenolecanium corni* from 2009 to 2013. (**a**) GLRaV-1, (**b**) GVA. *x*-axis: population density expressed as log (1 + *P corni* female number). *y*-axis: estimated probability of virus presence.

The relation between presence in vines of GLRaV-1 and GVA in the period 2009–2014 and *P. corni* numbers from 2009 to 2013 was variable and did not show any trend of increase with *P. corni* numbers (Figure 8). When the *P. corni* population was at its maximal density in 2007, mean number of females counted on vines before they became infected by GLRaV-1 (mean ± SD = 34 ± 32) or GVA (mean ± SD = 31 ± 29) in 2009–2014 was not significantly different from mean number of females on other vines (means ± SD for non-GLRaV-1- or -GVA-infected vines were identical: 36 ± 35, Student’s *t*-test, *p* > 0.05).

## 4. Discussion

Evolution of spatial distributions of *P. corni* females, as well as of GLRaV-1, GLRaV-3, and GVA viruses, were monitored from 2003 to 2015 in a commercial Riesling plot at Nothalten, northeast France. The plot was heavily infested by a *P. corni* population that grew from 2003 to 2007, and afterwards declined progressively up to 2015. The crop was conducted under organic pest management and no insecticide was used for at least 20 years. Parasitism [52] and predation [53,54] could have generated these fluctuations. In a study on the same plot from 2000 to 2005, parasitised females fluctuated annually between 39 and 76% [46,47,48,55], but it was not possible to determine whether these variations were the cause or the consequence of *P. corni* density changes. Conditions of relatively high temperature and humidity are beneficial to scale insect population growth [35,56,57], while low humidity [58] and extremes of summer and winter temperatures can affect them [59]. Cold tolerance experiments with *Parthenolecanium persicae* (Fabricius) showed that even at −15 °C, soft scales were still able to survive in dry conditions but were killed in wet conditions when temperatures reached −10 °C [60]. At Nothalten, annual extreme minimal or maximal temperatures did not seem to have an adverse effect on *P. corni* populations, even though the winter extreme in some years reached −10 °C (Figure 2). In the autumn, low temperatures leading to early leaf fall can cause a mortality of *P. corni* that can exceed 50% [61]. Thus, early low temperatures recorded in October from 2007 to 2009 could have contributed to the decline of *P. corni* populations. Among other factors affecting scale insect populations, heavy rain can reduce their abundance by dislodging eggs and nymphs from their hosts and favouring entomopathogenic fungi development [57,59,62]. Cultural management practices (fertilisation, pruning) can also influence scale insect abundance. High nitrogen levels in plants were shown to increase the reproductive performance of the citrus mealybug *Planococcus citri* (Risso) [63] and the vine mealybug *Planococcus ficus* (Signoret) [64]. Conversely, short pruning removes more soft scales [65,66]. In the case of our study, these conditions remained similar during the successive years of survey and were unlikely responsible for the high variation of density noticed.

The mapping of infested vines distribution, associated with a statistical spatial analysis of females, enabled us to assess the aggregated pattern of *P. corni*. The results of the present study agree with aggregated spatial patterns that have been reported for other mealybug species on grapevine [14,67]. Statistical spatial analysis also showed the aggregated pattern of GLRaV-1-, GLRaV-3-, and GVA-infected vines up to distances of 8, 4, and 6 intervals (1.40 m), respectively, between vine stocks within rows. For GLRaV-1 and GVA, distribution maps of newly infected vines showed that the contamination progressed around infected vines, mainly first to the closest neighbouring vines on the same row owing to the entanglement of canopies. Moreover, trellising wires provide pathways to soft scales and ants transporting nymphs between vines. Similar results were obtained with mealybugs in vineyards by several authors [10,18,20,51,68,69] who globally found that, among neighbours of infected vines, first nearest vines were most prone to becoming infected. Generally, virus infection spread primarily along the row, and to a lesser degree across rows. In our plot, GLRaV-1 and GVA distribution progressed from the limits of initial foci observed in 2003–2008. Distribution of viruses at plantation in the Riesling plot of Nothalten was unknown, since documented data about the history of the vine plot surveyed are lacking. The plot had been planted with massal selection vines, and some of these were probably infected and thus were at the origin of primary virus introduction. Contamination then progressed among stocks as expected due to insects, whose dispersal capacities are poor. Secondary spread seemed mainly due to *P. corni*, which was present at least once on almost all the stocks during our monitoring and whose distribution was aggregated. Moreover, no case of infection has been obtained after infectivity tests made in the laboratory with *P. vitis* and *H. bohemicus* nymphs sampled from the same plot [44].

Vine infection did not appear to depend on abundance of their natural vector. Presence in vines of GLRaV-1 and GVA in the period 2009–2014 and *P. corni* numbers in years from 2009 to 2013 did not show any trend of increase with *P. corni* numbers. When the maximal population density was reached in 2007, infection was independent of female numbers per vine, which was not different between future infected vines and healthy vines in 2009–2014. Similarly, Charles et al. [11] found no relationship between the spatial position of high mealybug infestations along old vine rows and the appearance of new GLRaV-3 infection in young neighbouring vines rows. Evidence of widespread clustering was only observed after a “high” mealybug year and when the young vines were large enough to sustain numbers of mealybugs. In our study plot, the discrepancy between the decline of *P. corni* population and virus spread explains difficulties sometimes encountered when searching for the origin of contaminations, for example, when insecticides were applied against scale insects in the meantime. When a plot becomes almost entirely contaminated, disease distribution becomes regular, making it difficult to find initial foci or a link with vector distribution.

The percentage of GLRaV-1- and GVA-infected vines grew by 24 and 12%, respectively, over a 12-year period. The expansion of the viruses was slow compared to that caused by mealybug species such as *Phenacoccus aceris* (Signoret) [14] or *Planoccocus* spp. [8,13,69], for which infection rates reached near 100% in the lapse of 5 to 10 years. Our infectivity tests with *P. corni* showed that transmission rates ranged from 28 (with 100 L1/recipient vine) to 41% (with 50–100 L2) for GLRaV-1, and from 26 (with 100 L1) to 36% (with 50–100 L2) for GVA (GH, unpublished). Moreover, *P. corni* nymphs are less mobile than mealybug nymphs, and adult females are sessile. This could explain the low transmission success of this soft scale species in the vineyard in comparison with the above-mentioned species. In Italy, virus spread due to *H. bohemicus* was also slow [70], and might also be linked to low transmission capacities. Bertin et al. [71] observed that although *H. bohemicus* nymphs showed high virus detection rates (88 to 100%), virus transmission occurred at lower rates (26%). Zorloni et al. [72] reported that *H. bohemicus* transmitted GLRaV-3 to 2 out of 77 inoculated test plants and GVA to 1 out of 38, whereas GLRaV-1 was not transmitted.

In our study, a few newly infected vines were recorded at over 4 m from previously infected vines. Nymphs could have been brought there by the wind, as observed with mealybugs [27,28] and with *P. corni* in a new planted vine adjacent to the present study plot [29,30]. The latter species may serve as a secondary disseminator of viruses, being able to colonise new plots with its wind-borne first instars. Counts of L2 settled in this young vine indeed showed the fast increase of *P. corni* populations [30]. 

On the basis of the present study, the low rate of virus spread despite a high density of *P. corni* population leads us to expect that, in an already infested and infected vine plot, removal of only infected vines could be sufficient to limit virus dispersal. *P. corni* represents a potential threat in infected vineyards due to dispersal of its crawlers by the wind. Planting new vineyards with virus-free material (rootstocks and cuttings) is necessary to prevent the expansion of GLRaV-1 and GVA, as *P. corni* is widespread in the vineyard and potentially in the surrounding environment. Planting non-host green hedges [73] and local control of high populations of scale insects could be further methods to limit their increase, after the monitoring of female density, as well as observing ants, which are a useful indicator of their presence as they are attracted by honeydew produced by scale insects.

## Figures and Tables

**Table 1:** Dates of first temperature ≤ 5 °C in October during the survey of *Parthenolecanium corni* female populations.

Date	31/10/06	8/10/07	5/10/08	3/10/09	19/10/10	16/10/11	8/10/12	11/10/13	24/10/14	12/10/15
Temperature	4.8 °C	5 °C	3 °C	5 °C	2 °C	1 °C	5 °C	4.5 °C	4 °C	5 °C

**Table 2:** Numbers of new infected vines in 2009–2014 according to their position around vines previously infected (I) in 2003–2008. I + 1: first neighbour within the same row, I + 2: second neighbour within the same row, O: opposite vine within the next row, O + 1: first neighbour of the opposite vine within the next row, O + 2: second neighbour of the opposite vine within the next row, B: beyond the preceding positions.

	Vine Position					
New Infected Vines	I + 1	O	I + 2	O + 1	O + 2	B
GLRaV-1	83	25	23	10	6	8
GVA	27	5	9	5	0	5
